# Supplementary material for: Predicting visual function by interpreting a neuronal wiring diagram
Source: Nature. 2024 Oct 2;634(8032):113–23. doi: 10.1038/s41586-024-07953-5 (PMC11446822; doi:10.1038/s41586-024-07953-5)
Supplement: Supplementary file 6 — CRF and ERF predictions for individual TmY4 and TmY9 cells. Analogous to Supplementary Data 3, but for TmY target types. Shown are the top four monosynaptic pathways, the strongest pathway passing through each of the top ten intermediary types (ranking from Extended Data Fig. 7), and the trisynaptic pathway Tm1–TmY–Dm3–TmY (see the section entitled Prediction of spatial normalization). [file 41586_2024_7953_MOESM6_ESM.zip › DataS4/TmY9qΓèÑ/720575940620060807.pdf]

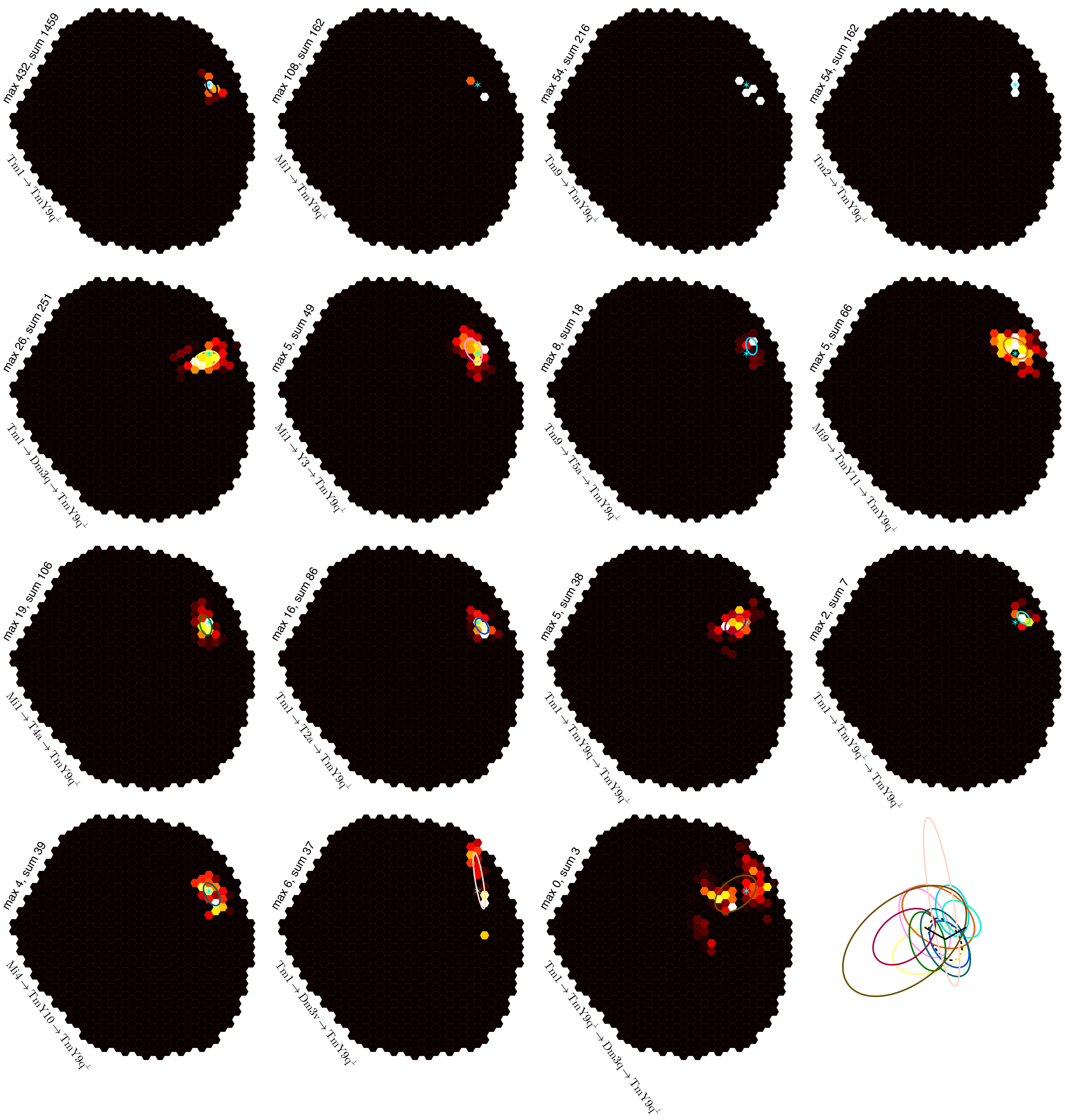
max 432, sum 1459
Tm1 → TmY9q⊥
max 108, sum 162
Mi1 → TmY9q⊥
max 54, sum 216
Tm9 → TmY9q⊥
max 54, sum 162
Tm2 → TmY9q⊥
max 26, sum 251
Tm1 → Dm3q → TmY9q⊥
max 5, sum 49
Mi1 → Y3 → TmY9q⊥
max 8, sum 18
Tm9 → T5a → TmY9q⊥
max 5, sum 66
Mi9 → TmY11 → TmY9q⊥
max 19, sum 106
Mi1 → T4a → TmY9q⊥
max 16, sum 86
Tm1 → T2a → TmY9q⊥
max 5, sum 38
Tm1 → TmY9q → TmY9q⊥
max 2, sum 7
Tm1 → TmY9q⊥ → TmY9q⊥
max 4, sum 39
Mi4 → TmY10 → TmY9q⊥
max 6, sum 37
Tm1 → Dm3v → TmY9q⊥
max 0, sum 3
Tm1 → TmY9q⊥ → Dm3q → TmY9q⊥
